# Disturbing Dreams and Psychosocial Maladjustment in Children: A Prospective Study of the Moderating Role of Early Negative Emotionality

**DOI:** 10.3389/fneur.2020.00762

**Published:** 2020-08-07

**Authors:** Aline Gauchat, Antonio Zadra, Mira El-Hourani, Sophie Parent, Richard E. Tremblay, Jean R. Séguin

**Affiliations:** ^1^Clinique de Consultation Conjugale et Familiale Poitras-Wright, Côté, Longueuil, QC, Canada; ^2^Department of Psychology, Université de Montréal, Montreal, QC, Canada; ^3^CHU Ste-Justine Research Center, Montreal, QC, Canada; ^4^School of Psychoeducation, Université de Montréal, Montreal, QC, Canada; ^5^Department of Pediatrics, Université de Montréal, Montreal, QC, Canada; ^6^School of Public Health, University College Dublin, Dublin, Ireland; ^7^Department of Psychiatry and Addictology, Université de Montréal, Montreal, QC, Canada

**Keywords:** disturbing dreams, nightmares, bad dreams, dreaming, psychosocial maladjustment, temperament, negative emotionality

## Abstract

Although frequent disturbing dreams, including bad dreams and nightmares, have been repeatedly associated with poor psychological well-being in adults, considerably less information exists on their psychosocial correlates in children. Recent empirical and theoretical contributions suggest that the association between disturbing dream frequency and psychosocial adaptation in children may differ as a function of children's negative emotionality. The current study assessed the moderating effect of very early negative emotionality (17 months of age) in the relation between disturbing dream frequency and psychosocial maladjustment (i.e., externalizing + internalizing behaviors) in a sample of 173 11-year-old children. Mixed-model analyses revealed that disturbing dream frequency was associated with some internalizing behaviors but that the association between disturbing dream frequency and most externalizing behaviors was moderated by early negative emotionality. The latter result indicates that the relation between disturbing dream frequency and externalizing behaviors was significant in 11-year-old children showing moderate negative emotionality early in life, but particularly strong in those children with high early negative emotionality. Whereas, a moderating effect of early negative emotionality was not found between disturbing dream frequency and internalizing behaviors, the findings highlight the more specific role of early emotional negativity as a developmental moderator for the link between disturbing dreams and externalizing behaviors in children. The results are discussed in light of recent models of disturbed dreaming production.

## Introduction

Up to 20% of children experience psychosocial adjustment difficulties (1, 2) which are typically divided between externalizing and internalizing problems (3, 4), although both categories evince common and unique risk factors (5). Studies show that while good sleep quality is associated with optimal behavior development, poor sleep is related to both externalizing and internalizing problems (6–17). Even after controlling for other risk factors, sleep problems appear to explain a small but significant proportion of the variance in both externalizing and internalizing behaviors (15, 18). However, different sleep problems appear to be associated with different externalizing or internalizing problems (6, 19).

Among these sleep problems, disturbing dreams (DDs; vivid dreams marked by intense negative emotions such as fear, anxiety, and anger) are frequently experienced by children (20–24). DDs may be associated with a wide range of psychosocial difficulties as they may signal problems with emotional regulation processes that normally occur during sleep as well as in dreams (for more details about this hypothesis, see (22, 25–28). While frequent DDs, including bad dreams and nightmares, have been repeatedly associated with poor psychological well-being and increased psychopathology in clinical and non-clinical adult populations (27), considerably less information exists on their psychosocial maladjustment correlates in children.

A recent review of the literature suggests that the occurrence of frequent DDs during childhood and adolescence is associated with a range of difficulties (21). First, DD frequency is associated with other sleep-related problems including insomnia, sleepwalking, bruxism, sleep talking, sleep terrors, nighttime awakenings, and unwillingness to go to bed (29–37). Second, DDs have also been linked to a range of specific mental health symptoms and psychosocial problems, including low prosociality (37, 38), academic problems (33), emotional excitability and being easily emotionally hurt (39), emotional symptoms (37, 38, 40), psychotic experiences (41, 42), borderline personality disorder (40), other mental health disorders (43), as well as suicidal ideation and suicide attempts (44–46). A prospective study found that DD frequency at 12 years of age was related to symptoms of psychosis during adulthood (47).

Moreover, DD frequency has been associated with internalizing problems in childhood and adolescence and repeatedly linked to anxiety (6, 48–54). Prospective studies of adolescents and late teenagers have similarly revealed links between parent-rated DDs at 10–19 years of age and later symptoms of anxiety and depression at 18–32 years of age (55). Moreover, DD frequency has also been linked to externalizing problems. Indeed, some studies of children show that DDs are associated with conduct disorders (37), rule-breaking, aggression, impulsivity (56), and hyperactivity (37, 57, 58). However, other studies did not find these associations (38, 59). Some studies also reported significant associations between DD frequency and attention deficit hyperactivity disorder (ADHD) (60) as well as with ADHD subtypes (61, 62), and one prospective study found that parent-rated DDs at 10–19 years of age predicted symptoms of inattention and direct/indirect aggression at 18–32 years of age (55).

Finally, some studies reviewed above combine sleep-related difficulties into one construct, thus not taking into account the differential relations of each sleep problem with unique measures of daytime functioning (16, 63, 64). Thus, whether or not DDs are related equally across internalizing and externalizing problems remains to be determined.

To further clarify inconsistencies in the literature, the construct of “affect distress,” defined as a trait-like disposition to react to emotional stressors with heightened negative affect and distress (27, 65) should be taken into account as it may play a role in the association between DDs and psychosocial adjustment difficulties. First, Levin and Nielsen's (27) model of DD production proposes that DDs occur out of an interaction between “affect load,” or day-to-day variations in emotional stress, and the aforementioned construct of affect distress, which can be viewed as a temperament subtype. Affect distress would fall under the negative emotionality dimension of temperament (66, 67). In the developmental literature, negative emotionality encompasses individual differences in typical reactions to negative emotional experiences, which can be readily observed from birth (68, 69). In fact, negative emotionality was found to be stable from early childhood to early adolescence (70) and was found to have a moderate continuity 17 years later (3–20 years old) (71).

Second, negative emotionality or difficultness, which refers to the tendency to experience negative emotions (fear, anger, sadness, discomfort) and high emotional distress when confronted to novel, ambiguous, and intense situations (66), has been related to psychosocial maladjustment throughout infancy and childhood (72–74). Recently, it is its moderating effect that has been mainly reported in the literature. Indeed, difficultness is already known to moderate relations between a variety of risk factors and children's behavior, such as the associations between cumulative contextual risk and children's externalizing, internalizing, and sleep problems (75–78). Some early temperamental characteristics may indeed predispose children to develop later behavior problems, particularly when other risk factors are present (79–82).

Taken together, Nielsen and Levin's (22) model of DD production and developmental literature on temperament suggests that the link between the frequency of DDs and psychosocial adaptation may differ as a function of a child's negative emotionality. This hypothesized moderator effect of negative emotionality has never been empirically tested. Furthermore, there is a lack of data on the relation between DDs and externalizing problems, and most studies of DDs in children suffer from one of the following three methodological limitations. First, DD frequency in children is often assessed through parent reports, despite the fact that this method has been shown to underestimate DD frequency and distress in comparison to child self-reports (37, 48, 50, 83). Second, many studies did not include any adjustment for possible confounding factors, such as risk factors common to both psychopathology and DDs (e.g., socio-economic status or comorbid sleep problems), despite the fact that taking such factors into account has been shown to attenuate observed associations (84). Third, and finally, although it is important to measure children's psychosocial maladjustment through multiple informants due to differences in child behavior across contexts (58, 85), multiple informants have rarely been used in the reviewed studies (21).

The goal of the present study was to first investigate the relationship between DD frequency and psychosocial maladjustment in children across a wide range of internalizing and externalizing behaviors and, second, to take into account the possible moderator effect of early emotional negativity. Methodological shortcomings characteristic of many studies in the field were also addressed: (a) measures of DD frequency were obtained from the children themselves, (b) socio-economic status and co-morbid sleep disorders were taken into account in the analyses, and (c) children's psychosocial maladjustment was assessed through multiple informants.

Two main and complementary predictions were tested: (1) DD frequency will be positively associated with psychosocial maladjustment across several internalizing and externalizing behavioral domains; and (2) negative emotionality will moderate this relation between measures of DD frequency and psychosocial maladjustment across several internalizing and externalizing behavioral domains. Specifically, we tested the hypothesis that the association between DD frequency and internalizing and externalizing problems would be strengthened with increasing levels of negative emotionality. These hypotheses will be tested separately for each internalizing (anxiety, social withdrawal, and emotional problems) and externalizing (opposition, physical aggression, reactive, proactive aggression, indirect aggression, and ADHD) symptoms.

## Materials and Methods

### Participants

Participants were part of a longitudinal study focusing on the social, psychological, and cognitive development of children from urban socio-economic backgrounds in the province of Québec, Canada. At the study's inception, 1,000 families were randomly selected from the Québec 1996–1997 birth register (86). Of these, 572 consented to participate in the original study and were then assessed annually in French (~82%) or English (Canada's two official languages) from the age of 5 months. Due to annual attrition, variability in the participants' year to year availability for data collection, and funding constraints which limited the capacity to follow-up all families, a total of 173 children (comprised equally of boys and girls) completed the present study (mean age = 11.4 years, SD = 0.1). These 173 children did not differ from the remainder of the original 572 5-month-old children in terms of their socio-economic level, including family income (*p* = 0.68), family type (single parent or not; *p* = 0.57), maternal level of education (*p* = 0.33), or negative emotionality at 17 months of age (*p* = 0.54). They did not differ on other behavioral measures at 17 months in term of hyperactivity (*p* = 0.73); inattention (*p* = 0.89); emotional troubles (*p* = 0.77); anxiety (*p* = 0.74); physical aggression (*p* = 0.71), except for opposition with children in the current sample being a little less oppositional (M = 3.2; SD = 1) than the remainder of the sample (M = 3.5; SD = 1.1); *t* = 2.8; *p* < 0.05.

### Measures

#### Assessment of Disturbing Dreams

Participants from this longitudinal sample self-reported about their DDs for the first time at 11 years of age. The instructions to children referred to DDs using the expression “bad dreams” (defined as very disturbing dreams) because the term DD was too unfamiliar to them given their age. Children were required to answer the question: “On average, how frequently do you have bad dreams?” using the following choices: “Never,” “Sometimes,” “Often,” “Always,” or “Don't know.” Participants reporting bad dreams were also asked to estimate the number of bad dreams experienced over the past month. For children who reported “never” in the previous question, the number of bad dreams was coded as 0 while maximum frequency was set at 30 (i.e., 1 DD/day) to limit the impact of potential outliers. Thus, values for monthly DD frequency ranged between 0 and 30. A 1-month retrospective frequency estimate was used instead of a 1-year estimate as it has been shown to correspond more closely to prospectively collected log-based frequency measures of DDs from the same individuals (87, 88).

#### Measures of Psychosocial Maladjustment

Psychosocial maladjustment was measured using a battery of validated scales (89–91) drawn from various instruments. Some scales from the Preschool Behavior Questionnaire (92), the Child Behavior Checklist (93), and the Reactive and Proactive Aggression Questionnaire (94) were used to create the questionaire. The scales (and mean Cronbach alpha across informants) included measures of both internalizing problems: anxiety (4 items, Mα = 0.72, e.g., being nervous, high-strung or tense), social withdrawal (3 items, Mα = 0.68, e.g., prefers to play alone rather than with other children), and emotional problems (3 items, Mα = 0.69, e.g., has trouble enjoying him or herself); and externalizing problems: opposition (3 items, Mα = 0.51, e.g., punishment doesn't change the child's behavior), physical aggression (4 items, Mα = 0.75, e.g., physically aggresses people), reactive aggression (4 items, Mα = 0.75, e.g., reacts aggressively when someone takes a personal belonging, for example by hitting, pushing, or slapping another child), proactive aggression (3 items, Mα = 0.55, e.g., scares other children to get what is wanted), indirect aggression (3 items, Mα = 0.69, e.g., when angry at someone, tries to get others to dislike the other person), and ADHD symptoms (7 items, Mα = 0.86, e.g., cannot settle on anything for more than a few moments; is impulsive/acts without thinking; is inattentive). These validated scales (89, 91) have been shown to be sensitive to various environmental, familial, and perinatal risk and protective factors (95–99) as well as to early sleep patterns (100). The instrument was completed by the participants themselves at 11 years of age as well as by each child's father and teacher in order to get a complete description of their difficulties across the social context. Questions for the child version were read to them by the research assistant but they could record answers confidentially.

### Covariables

#### Socio-Economic Status

Three variables were used to evaluate each family's socio-economic status: family income (continuous variable), level of maternal education (dichotomous variable, with a high level being defined as having a secondary school diploma or higher), and whether or not the child was in a single-parent family (dichotomous variable).

#### Sleep

Two sleep-related variables were included: sleepwalking, since this sleep disorder has been repeatedly associated with DDs (29, 33, 34, 39, 57) and daytime somnolence because poor sleep quality (which usually leads to daytime somnolence) is associated with poor mood and behaviors (6, 100, 101). Two questions to the mother were “Does your child sleepwalk in his/her sleep?” and “In general, is your child sleepy during the day?” Both could be answered with “Never,” “Sometimes,” “Often,” or “Always.”

#### Negative Emotionality

Negative emotionality was assessed at 17 months using a shortened scale developed by Vitaro and colleagues (102) of the original fussy/difficult temperament scale developed by Bates and colleagues (103) (example of item: intensity of the child's protest). This seven-item scale was completed by the mother, in order to avoid a shared-method variance problem with the informants who completed the psychosocial adjustment measures for our analyses (102). The measure of negative emotionality used in the present study showed good internal consistency with a Cronbach alpha of 0.71.

### Procedure

Each parent, or legal guardian responsible for the child, and teacher received an invitation by mail to participate in the study. Consent was obtained from parents or legal guardians and assent was obtained from the child. The study was approved by the Research Ethics Committee of the CHU Ste-Justine Research Center, and the study protocol also complied with the ethical guidelines of the American Psychological Association (“Ethical Principles of Psychologists and Code of Conduct,” 2017).

### Analyses

Model used: Mixed-effects model analyses were estimated using the “Statistical Package for the Social Sciences” (Version 18) (104) to investigate relationships between DDs and dimensions of psychosocial maladjustment. Nine models were tested, one for each measure of psychosocial maladjustment, three for internalizing problems, and six for externalizing problems. Specifically, responses provided by the three informants (child, teacher, and father) were included for each psychosocial maladjustment scale within the mixed-effects models where each of the informants represents a repeated component. A mixed-effects model as opposed to a traditional repeated-measure ANOVA allows for an unbalanced design. This model is also adapted for situations with unequal covariance as it does not assume equal correlations across informants. In a mixed-effects model, if one or two informants are missing, the other informants are still included in the analyses, whereas traditional repeated-measures ANOVA requires data from all three informants for each participant entered in the analyses. Thus, the mixed-effect model actually uses all the available information for parameter estimation (105). Responses were available from all three informants for 78 participants (42.6%)—from the child and teacher for 38 participants (22%), from the child and father for 32 participants (18.6%), and only from the child in 32 cases (18.6%). There was no systematic pattern of missing responses related to child behavior outcomes.

#### Preliminary Analyses

Sex differences on DD frequency measures were assessed with *T*-tests. Potential a priori covariates were selected for inclusion in the analyses on an empirical basis in order to enter only meaningful variables and avoid loss of power (106) as it has been done in other studies (81). This was done by first computing correlations or ANOVAs between potential control variables (socioeconomic status; SES, sleepwalking, somnolence) and outcome variables to determine which control variables should be entered as fixed effects for which outcome measures. If a potential control variable was related to an outcome variable (at *p* ≤ 0.05) assessed by at least one of the informants, it was entered in the analysis. Maternal level of education was thus entered in the analyses for anxiety, physical aggression, ADHD symptoms, and opposition, whereas family income was entered in the analyses for anxiety, ADHD symptoms, and opposition (for all those variables, a higher socio-economic status was linked to less psychosocial maladjustment). The family type and sleep disorder variables were not related to any of the psychosocial maladjustment measures. Whenever interactions between two variables were not significant, the interaction term was removed from the analytical model and only the main effect was tested.

Our two overarching hypotheses were tested using the mixed-effects model for each psychosocial maladjustment problem within internalizing and externalizing problems. Informant source and child sex were always entered as fixed effects. Interactions between DD measures and child sex or informant source were tested since children's behaviors can be perceived differently at school vs. at home and because behavioral difficulties differ between boys and girls (85, 107, 108), resulting in differences in observed associations between DDs and behavioral difficulties as a function of context and sex.

Interactions between DDs and early negative emotionality were investigated to test the second hypothesis which proposed that early negative emotionality moderates the relationship between DD frequency and psychosocial maladjustment. DD frequency and early negative emotionality were centered.

## Results

### Descriptive Statistics

Of the 173 children that completed the questionnaires, 129 (82.7%) reported experiencing at least one DD per month. The mean frequency of DDs reported per month by the entire sample was 3.6 (*SD* = 5.3), with 12% of the sample having 10 or more DD per month. A significantly greater proportion of girls (88.7%) reported having at least one DD in the last month than did boys (76.3%), χ^2^ = 4.21, *p* < 0.05. However, there were no significant sex differences in the actual number of DDs experienced in the past month. Using the MIXED models, none of the DDs by informant interactions were significant, and early negative emotionality was not correlated to DDs (*r* = 0.04; *p* = 0.58). Correlations between each informant for a given variable vary between *r* = 0.03 for opposition and *r* = 0.28 for anxiety with a mean of M*r* = 0.17.

Before testing the moderating effect of early negative emotionality on each childhood-dependent variable, we examined its correlation with DDs to determine if it met the criteria for moderation analysis. DDs and early negative emotionality were not correlated (*r* = 0.04; *p* = 0.58). We also examined associations between early negative emotionality and dependent variables. Correlations ranged from *r* = 0.03 to *r* = 0.07 with a mean of M*r* = 0.05 for internalizing behaviors, and they ranged from *r* = 0.05 to *r* = 0.20 with a mean of M*r* = 0.09 for externalizing behaviors. None of those correlations were significant except the one between early emotional negativity and physical aggression (*r* = 0.19; *p* ≤ 0.05).

### Associations Between DD Frequency and Internalizing Behaviors

When considering internalizing behaviors, none of the interactions between DDs and temperament were significant (all *p*_s_ > 0.50). They were therefore removed from the models. Table 1 shows main effects and effect sizes for the association between DD frequency and internalizing problems once control variables were included in the statistical models. DD frequency was positively related to 2 of the 3 measures of internalizing behaviors (social withdrawal and emotional problems), with Cohen's *d* statistics of effect size for continuous variables in the small to medium range (109). This only partially supports the first hypothesis, as no moderating effect of early negative emotionality was found.

**Table 1:** Main effects of DD frequency on internalizing problems.

**Variables**	***F***	***df(v1, v2)***	***p***	***d***
Social withdrawal	5.26	1,141.75	0.02	0.39
Emotional problems	9.17	1,175.91	0.003	0.45
Anxiety	2.89	1,131.75	0.09	0.30

### Associations Between DD Frequency and Externalizing Behaviors

**Figure 1:**
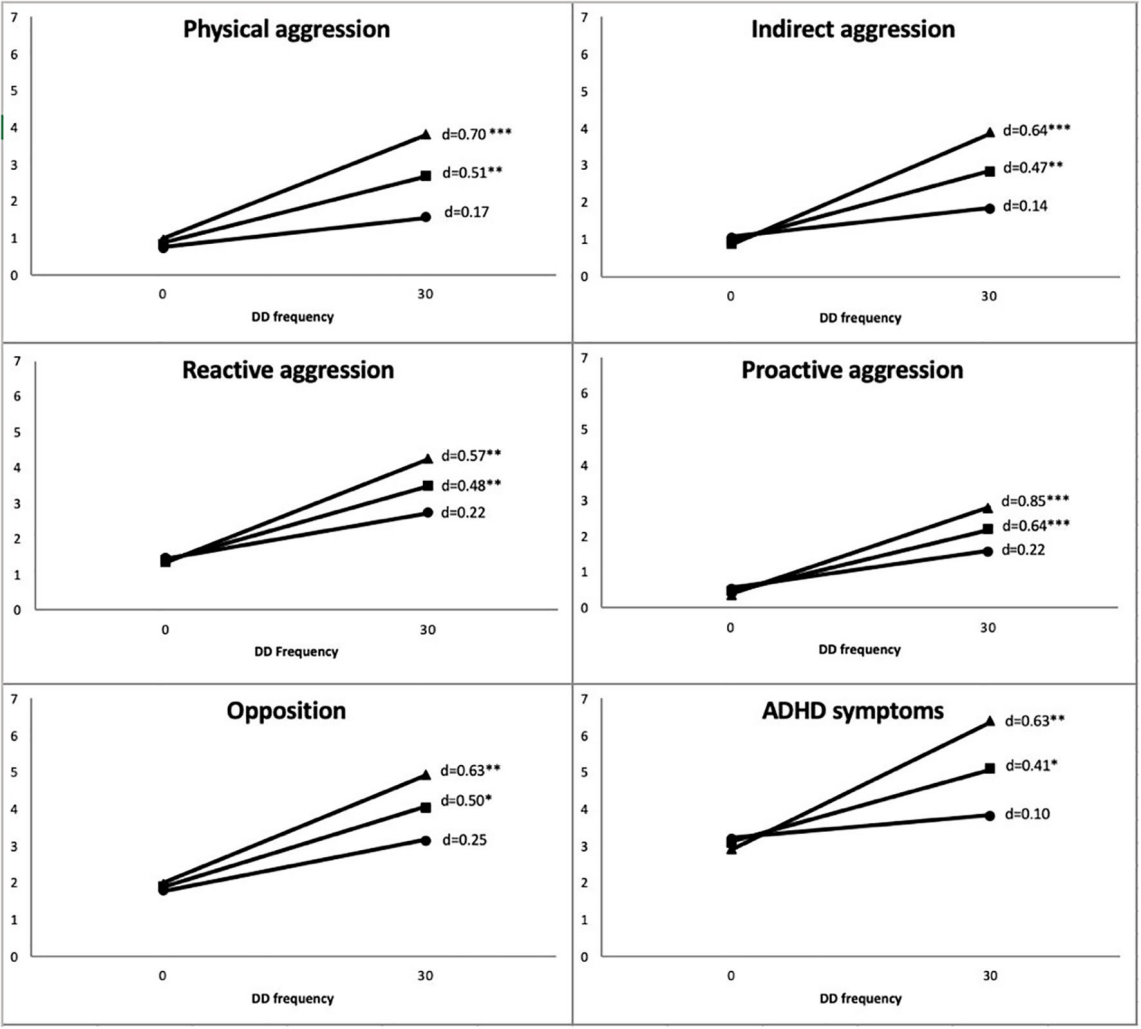
Strength of association between monthly DD frequency (x-axis) and externalizing behaviors (y-axis) as a function of averaged negative emotionality and ± 1 SD. *p < 0.05; **p < 0.01; ***p < 0.001. Externalizing behaviors were standardized across a range from 0 to 7. Monthly DD frequency ranged from 0 to 30.

The interaction term between early emotional negativity and DDs was kept in all models examining externalizing behaviors because *p*-values were consistently below 0.13 (ranging from 0.001 for proactive aggression and 0.13 for opposition), suggesting that this pattern was not random. Table 2 presents results of the analyses of main effects and interactions for each externalizing behavior. Figure 1 plots these interactions using the method proposed by Aiken and West (110) by showing the strength of the association between DD frequency and each dimension of externalizing behaviors plotted as a function of early negative emotionality level [low (−1 SD), average or high (+1 SD)]. As can be seen in Figure 1, the pattern of interactions was highly consistent across dimensions of externalizing behaviors, where associations between frequency of DDs and externalizing behaviors were systematically positive and significant for children with moderate to high levels of early negative emotionality. For these children, effect sizes were medium to large, ranging from *d* = 0.41 to *d* = 0.85 (109). This pattern of interactions and absence of main effects only partially supports the second hypothesis.

**Table 2:** Main effects of DD frequency and interactions with early negative emotionality for each externalizing behavior problem.

	**Main effects of DDs**				**Main effects of negative emotionality**				**Interaction between DDs and negative emotionality**			
**Variables**	***F***	***df(v1, v2)***	***p***	***d***	***F***	***df(v1, v2)***	***p***	***d***	***F***	***df(v1, v2)***	***p***	***d***
Indirect aggression	0.85	1,160.9	0.36	0.14	0.59	1,160.1	0.44	0.12	6.64	1,154.8	0.01	0.41
Physical aggression	0.68	1,136.9	0.41	0.14	0.48	1,125.1	0.49	0.12	6.21	1,129.1	0.01	0.44
Reactive aggression	0.42	1,160.2	0.52	0.10	0.36	1,154.7	0.55	0.09	4.25	1,152	0.04	0.33
Proactive aggression	0.10	1,140.8	0.33	0.05	1.69	1,138.2	0.19	0.22	9.41	1,134.1	0.003	0.53
Hyperactivity, impulsivity and attention symptoms (HIA)	1.03	1,129.9	0.31	0.17	0.51	1,118.2	0.48	0.13	5.69	1,120.1	0.02	0.44
Opposition	0.01	1,127.9	0.97	0.02	0.34	1,114.9	0.56	0.10	2.34	1,117.5	0.13	0.28

## Discussion

The results of the present study partially support our predictions that DD frequency would be positively associated with psychosocial maladjustment in children and that early emotional negativity would moderate this relationship. In line with previous adult and childhood literature, DD frequency was positively related to psychosocial maladjustment of the internalizing type: across informants, children with higher DD frequency scored higher on two of the three measures of internalizing problems (i.e., emotional problems and social withdrawal) without moderation by early emotional negativity. For psychosocial maladjustment of the externalizing type, there was a moderation effect of early emotional negativity: the association between DDs and externalizing difficulties was stronger with increasing levels of early emotional negativity.

The associations between DD frequency and internalizing behaviors remained significant even after adjusting for socio-economic and sleep-related variables. That DDs in children were associated with a range of internalizing problems (e.g., emotional problems and social withdrawal) is consistent with findings reported in children and adult populations (21, 27, 40, 56). However, unlike some reports, DD frequency in the present study was not significantly related to anxiety. The effect size for this relation (*d* = 0.30) was too small to reach statistical significance given our sample size. By comparison, effect sizes in previous studies have ranged between 0.20 and 0.72 (M*d* = 0.53) (49, 51, 53). Differences in measures of anxiety themselves (e.g., source of informant, symptoms scale vs. clinical diagnosis) may also partially account for this. The fact that none of the interactions with informants were significant shows that despite contextual differences in behaviors, the association between DDs and psychosocial maladjustment is robust and did not differ as a function of informant source.

The absence of the hypothesized moderator effect of early emotional negativity on internalizing problems may have been related to the measure used in the present study. Specifically, our measure of early emotional negativity at 17 months may not have been sensitive to the complete spectrum of manifestations of affect distress. For example, in contrast to observations in infants, studies have shown that toddlers high in emotional reactivity are more likely to manifest their reactivity through inhibition or withdrawal (111–114). These behavioral manifestations have been associated with later proneness to internalizing difficulties (79, 115–117). By contrast, our measure did not include items specifically formulated to detect these behavioral manifestations of early emotional reactivity, which might explain why it did not correlate with, or moderate, later internalizing difficulties. Thus, our measure may be more sensitive to behavioral manifestations of toddlers' “affect distress,” like aggression, that are linked to later externalizing problems than to subsequent internalizing problems.

The finding that early negative emotionality specifically moderated the relationship between DDs and externalizing behaviors is new. In fact, this is the first study to document that DDs in children with early moderate to high early emotional negativity are strongly associated with externalizing behaviors (with corresponding ds ranging between 0.60 and 0.90). The consistency of results across externalizing problems may be due to a common underlying factor (3, 118). How the relation between frequent DDs and externalizing behaviors in children with a history of moderate to elevated negative early emotionality evolves over time remains to be clarified. However, some studies have shown that early emotional negativity may moderate other associations implicating later externalizing behaviors, such as the relation between early child care and externalizing behaviors during adolescence (119). Another study (76) found that low early negative emotionality at 18 months emerged as a protective factor in children experiencing a cumulative risk of developing internalizing and sleep problems at 24 months. Consequently, and in the absence of a concurrent measure of negative emotionality, this long-term effect noted across several studies also supports the hypothesis that negative emotionality is a relatively stable developmental characteristic.

The correlational nature of our study does not allow us to draw conclusions about the direction of the link between DDs and psychosocial adjustment. On the one hand, it is possible that DDs have an impact on psychosocial adjustment as it has been shown that DDs could influence the dreamer's mood the following day (120). Frequent DDs in children could similarly result in negative emotions and distress during wakefulness much like the nightmare-related distress documented in adults (27, 121–123). Thus, it is possible that repeated and negative experiencing of DDs elicits negative reactive emotions during wakefulness.

On the other hand, it is also possible that psychosocial adjustment problems and related perceived stress may have an impact on the frequency of DDs (27, 124, 125). In this case, DDs would reflect issues and concerns experienced during wakefulness. Alternatively, it is possible that a third variable explains the relation between DDs and psychosocial adjustment. This would be consistent with suggestions that DDs represent a failure in the emotional regulation function believed to occur during normal dreaming (126–128). Psychosocial maladjustment problems are also known to be related to problems in emotional regulation (74, 129–131). As discussed earlier, this interpretation would be consistent with the hypothesis of a moderator effect of emotional negativity and DDs on psychosocial adjustment. While our study has some key strengths including the use of an early measure of negative emotionality, control variables, and multiple informants for the assessment of psychosocial maladjustment, it also has some shortcomings. With the exception of the children's early emotional negativity measure, this was essentially a cross-sectional correlational study, and as such, it cannot address the developmental sequence with regard to DDs and psychosocial maladjustment.

Longitudinal studies are needed to clarify the nature and time course of DDs in relation to psychosocial maladjustment. Also, as has been done in adults, the inclusion of measures of potentially more severe forms of maladjustment such as suicidality (which emerges in early adolescence) may be helpful as suicidal ideation has been linked to both internal distress and externalizing behaviors such as impulsivity and conduct disorders (132–134). In fact, one review found that impulsivity and emotional dysregulation could explain the link between sleep disturbances and suicidality (135). More studies should investigate these associations specifically with DDs. In addition, the study of other moderator variables could further our understanding of these complex developmental issues. Disorganized attachment, for instance, is a promising candidate as it has been related to both externalizing behaviors and DDs and could play a moderator role in these relations (136–139).

In sum, the present study highlights the role of early emotional negativity as a developmental moderator for the link between DDs and externalizing behaviors in children and points to the need to consider temperamental traits when investigating associations between DDs and internalizing and externalizing problems from a developmental perspective.

## Data Availability Statement

Data may be available by completing a request on our website http://gripinfo.ca/grip/public/www/etudes/en/dadprocedures.asp?langue=en. All data requests are subject to current privacy laws guiding their ethical use in Québec, Canada, and examined by the CHU Ste-Justine Research Ethics committee.

## Ethics Statement

The studies involving human participants were reviewed and approved by Research Ethics Committee of the CHU Ste-Justine Research Center. Written informed consent to participate in this study was provided by the adult informants themselves, the study participants' parents or legal guardian, and assent was provided by the children themselves.

## Author Contributions

AG contributed to study design, analyzed the results, and wrote the manuscript draft. AZ obtained supporting grants, supervised the study, contributed to the statistical analyses, and reviewed the manuscript. ME-H reviewed the data collection and reviewed the manuscript. RT obtained supporting grants and contributed to the study design, data collection, and reviewed the manuscript. SP and JS obtained supporting grants, designed and supervised the study, contributed to the statistical analyses and their interpretation, and reviewed the manuscript. All authors contributed to the article and approved the submitted version.

## Conflict of Interest

The authors declare that the research was conducted in the absence of any commercial or financial relationships that could be construed as a potential conflict of interest.
